# Supplementary material for: Prediction model and risk scores of ICU admission and mortality in COVID-19
Source: PLoS One. 2020 Jul 30;15(7):e0236618. doi: 10.1371/journal.pone.0236618 (PMC7392248; doi:10.1371/journal.pone.0236618)
Supplement: Attachment — Submitted filename: response-to-reviewer.docx [file pone.0236618.s001.docx]

**Thank you for your careful review of our manuscript**

Reviewers' comments:

Reviewer's Responses to Questions

**Comments to the Author**

1. Is the manuscript technically sound, and do the data support the conclusions?

The manuscript must describe a technically sound piece of scientific research with data that supports the conclusions. Experiments must have been conducted rigorously, with appropriate controls, replication, and sample sizes. The conclusions must be drawn appropriately based on the data presented.

Reviewer #1: Yes

Reviewer #2: Yes

2. Has the statistical analysis been performed appropriately and rigorously?

Reviewer #1: Yes

Reviewer #2: Yes

3. Have the authors made all data underlying the findings in their manuscript fully available?

The [PLOS Data policy](https://nam03.safelinks.protection.outlook.com/?url=http%3A%2F%2Fwww.plosone.org%2Fstatic%2Fpolicies.action%23sharing&data=02%7C01%7CTim.Duong%40stonybrookmedicine.edu%7C2e18ca7c33244fbf53fe08d81faca053%7Ceafa1b31b194425db36656c215b7760c%7C0%7C0%7C637294173721358760&sdata=vaaJpH2rgLoxiHcEj%2F74dYEocQTeU%2FRbqLno27%2B8DJw%3D&reserved=0) requires authors to make all data underlying the findings described in their manuscript fully available without restriction, with rare exception (please refer to the Data Availability Statement in the manuscript PDF file). The data should be provided as part of the manuscript or its supporting information, or deposited to a public repository. For example, in addition to summary statistics, the data points behind means, medians and variance measures should be available. If there are restrictions on publicly sharing data—e.g. participant privacy or use of data from a third party—those must be specified.

Reviewer #1: No

Reviewer #2: Yes

4. Is the manuscript presented in an intelligible fashion and written in standard English?

PLOS ONE does not copyedit accepted manuscripts, so the language in submitted articles must be clear, correct, and unambiguous. Any typographical or grammatical errors should be corrected at revision, so please note any specific errors here.

Reviewer #1: Yes

Reviewer #2: Yes

5. Review Comments to the Author

Reviewer #1: This study aimed at developing risk scores based on clinical characteristics at presentation to predict ICU admission and mortality in COVID-19 patients. The paper is well written and show interesting data. However, the authors report just the AUCs of scores. Sensitivity, specificity and likelihood ratios of the proposed scores must be given.

**Thank you.**

**The sensitivity and specificity of the risk scores were 10.5% and 99.2% for predicting ICU admission, and 7.1% and 100% to predict mortality. The high specificity and low sensitivity were due to the imbalance of sample sizes where there were more patients in general admission group compared to ICU admission or death group.**

Reviewer #2: In this article by Dr. Timothy Duong et al they aim to develop risk scores based on clinical characteristics at presentation to predict intensive care unit (ICU) admission and mortality in COVID-19 patients. According to the authors, five significant variables predict ICU admission (lactate dehydrogenase, procalcitonin, pulse oxygen saturation, smoking history, and lymphocyte count) and seven variables predict mortality (heart failure, procalcitonin, lactate dehydrogenase, chronic obstructive pulmonary disease pulse oxygen saturation, heart rate, and age). The model predicts ICU admission and a mortality with an AUC of 0.74 and 0.83, respectively. This article is well written and addresses a very important issue regarding patient flow organization and allocation of resources in the COVID-19 era.

My main comments on the manuscripts have been addressed by the authors in the limitation section. Im looking forward to a follow-up study were authors add more institutions and make the study more generalizable.

I believe this article deserves to be published.

**Thank you.**

LINE 40: There is a comma missing between COPD and Pulse oxygen

**Thank you**
